# LINC01087 Promotes the Proliferation, Migration, and Invasion of Thyroid Cancer Cells by Upregulating PPM1E

**DOI:** 10.1155/2022/7787378

**Published:** 2022-03-25

**Authors:** Ying Yin, Jianhao Huang, Hongyan Shi, Yijie Huang, Ziyang Huang, Muye Song, Liping Yin

**Affiliations:** ^1^Department of General Surgery, Guangdong Provincial People's Hospital, Guangdong Academy of Medical Sciences, Guangzhou, Guangdong, China; ^2^Shantou University Medical College, Shantou, Guangdong, China; ^3^The Second School of Clinical Medicine, Southern Medical University, Guangzhou, Guangdong, China; ^4^School of Medicine, South China University of Technology, Guangzhou, Guangdong, China; ^5^Imaging Department, Shandong Cancer Hospital and Institute, Shandong First Medical University and Shandong Academy of Medical Sciences, Jinan, Shandong, China

## Abstract

This study is aimed at investigating the effect and mechanism of LINC01087 on the malignant evolution of thyroid cancer cells. The expression levels of LINC01087, miR-135a-5p, and PPM1E in thyroid carcinoma tissues were detected by QRT-PCR. Cell viability was detected using the CCK-8 method. Transwell assay was used to assess the ability of cells to invade. The targeting relationship between LINC01087 and miR-135a-5p was detected by dual luciferase reporting assay. In comparison with normal thyroid tissues and cells, the expression level of LINC01087 in thyroid cancer tissues and TPC-1 and K1 cells increased, and the expression level of miR-135a-5p in thyroid cancer tissues and TPC-1 and K1 cells decreased. LINC01087 knockdown and the high expression of miR-143-3p inhibited the proliferation, invasion, and EMT processes of TPC-1 and K1 in thyroid cancer cells. LINC01087 negatively targeted miR-135a-5p. Has-miR-135a-5p inhibited the malignant evolution and EMT of thyroid cancer by targeting PPM1E. The PPM1E overexpression can reverse the inhibitory effect of LINC01087 gene knockdown on the proliferation, migration, and invasion of thyroid cancer cells. LINC01087 can promote the proliferation and apoptosis of thyroid cancer cells, and its mechanism may be related to the miR-135a-5p/PPM1E axis.

## 1. Introduction

In recent years, the incidence of thyroid cancer is the fastest growing among all malignant solid tumors [1–3]. The occurrence of tumor results from a combination of environmental and genetic factors [4]. Changes in the environment can induce abnormalities, mutations, and deletions of tumor genes and tumor suppressor genes in the body, resulting in a group of similar physiological functions [5]. The new revolutionary technology characterized by the early diagnosis and targeted therapy of tumor genes has shown its unique superiority in tumor diagnosis and treatment [6].

Noncoding RNAs regulate development, differentiation, and metabolism [7]. LncRNA is closely related to many diseases [8]. The specific pathogenesis of thyroid cancer remains unclear. At the epigenetic level, the expression level of a specific type of noncoding RNA in thyroid cancer and the relationship between specific genes and signal transduction pathways related to the occurrence and development of thyroid cancer should be studied [9]. Identification and in-depth study from the molecular level is important to elucidate the occurrence and development mechanism, prevention, and early diagnosis and treatment of thyroid cancer [10]. LINC01087 is highly expressed in breast cancer. Mechanistic studies have shown that LINC01087 regulates the malignant behavior of cancer cells through the miR-335-5p/Rock1 axis [11]. In addition, Chen et al. reported that the LINC01087 expression is associated with poor preoperative MRI prognosis in patients with glioma [12]. The role of LINC01087 in thyroid cancer has not been reported.

miRNA can regulate the physiological and pathological processes of the body by regulating cell proliferation and apoptosis [13]. miRNA is abnormally expressed in ovarian cancer and can regulate cell proliferation, apoptosis, and other biological processes [14]. The upregulation of the miR-135a-5p expression can participate in biological processes, such as atherosclerosis and nerve cell injury [15, 16]. Reversible protein phosphorylation plays an important role in regulating protein activity and is involved in almost every major physiological process. Protein phosphatases are enzymes that catalyze the dephosphorylation of phosphorylated protein molecules. They correspond to protein kinases, which constitute the on-off system of phosphorylation and dephosphorylation, an important protein activity. Magnesium-ion-dependent protein phosphatase 1E (PPM1E) is a member of the serine/threonine protein phosphatase family [17, 18]. This family is considered to be an essential negative regulator of eukaryotic stress response pathways. Limited studies have reported the role of PPM1E in thyroid cancer.

The effects of LINC01087 and miR-135a-5p on the proliferation and invasion of thyroid cancer cells and their mechanisms remain unclear. This study investigated the effects of LINC01087 in thyroid cancer cells and whether LINC01087 could affect the proliferation and invasion of thyroid cancer cells by regulating miR-135a-5p and PPM1E. This study may provide new biomarkers and new targets for the prevention, treatment, and prognosis of thyroid cancer.

## 2. Methods

### 2.1. Thyroid Cancer Specimens Were Collected

Thirty cases of fresh frozen tissues from patients with thyroid diseases admitted to the Department of General Surgery of our hospital from January 2020 to January 2021 were selected. Paracancer tissue > 2 cm away from the tumor edge (no cancer cells under the microscope) and paracnodule tissue > 2 cm away from the benign nodular edge were selected as controls. All cases must meet the following criteria: (1) the patient is not associated with other tumors, (2) no serious organ failure such as liver and kidney failure, and (3) no history of chemotherapy or immunotherapy prior to surgery. The patient age ranged from 19 to 77. The median age was 54 years. A total of 7 males and 23 females were involved. All patients signed informed consent forms. This study was approved by our ethics Committee of Shandong Cancer Hospital and Institute.

### 2.2. Cell Culture and Transfection

Thyroid cancer cells TPC-1 and K1 were cultured in DMEM complete medium in a constant temperature incubator with 5% CO_2_ at 37°C. When the cell growth density reached approximately 80%, the subculture was carried out. Logarithmic ovarian cancer cells were inoculated into 96-well plates (3 × 10^4^ cells/well). Refer to the Lipofectamine 2000 reagent instructions to transfect sh-NC, sh-LINC01087, mimics-NC, Has-miR-135a-5pmimics, pcDNA3.1, and pcDNA3.1-PPM1E into cells. The culture was continued for 48 h, and the cells were collected. Untransfected ovarian granulosa cells were used as the NC group.

### 2.3. CCK-8

The cells were planted in 96 empty plates with 1 × 10^3^ cells/well. Each group had three multiple holes. Cells were cultured in 100 *μ*L of medium per well. At 72 h after transfection, 10 *μ*L of CCK-8 reagent was added and incubated at 37°C for 2 h. The absorption value of 450 nm wavelength was detected, and the average value of three complex holes was obtained. The absorbance value at each time point was connected to draw the cell proliferation curve.

### 2.4. EdU

The transfection group was the experimental group, and the transfection of no-load plasmid was the control group. At 48 h after transfection, cell proliferation was detected according to the instructions of EdU kit. The nuclei were restained by DAPI and photographed under fluorescence microscope. The ratio of EdU-positive cells to DAPI-positive cells was used to analyze cell proliferation.

### 2.5. Transwell

Single-cell suspension of transfected cells was prepared using trypsin method at 48 h after transfection. Approximately 5 × 10^4^ transfected cells were suspended in fetal bovine serum DMEM. Matrigel (1 : 3) was added to the upper chamber of the transwell experiment. The sample was inoculated into the upper compartment of the 24-well transwell compartment. The chamber is equipped with a polycarbonate film filter (aperture, 8 *μ*m). Matrigel was added early in the upper chamber. The transplanted cells were fixed and then stained with 0.5% crystal violet for 30 min.

### 2.6. qRT-PCR

The TRIzol method was used to extract the required RNA product. The extracted thyroid cancer tissue or cell RNA was reverse transcribed to obtain cDNA. Real-time fluorescent quantitative PCR with SYBR green underwent predenaturation at 95°C for 5 min, 95°C for 10 s, 58°C for 45 s, and 72°C for 20 s for 50 cycles. The reaction system was configured according to the kit instructions and placed on a PCR instrument to detect the Ct value of each gene. miRNA used U6 as the internal control, and PPM1E used *β*-actin as the internal control. The corresponding Ct value was recorded, and the 2^-*ΔΔ*Ct^ method was used for quantitative analysis (Table 1).

### 2.7. FISH Assay

A 4 *μ*m-thick tissue section of the tumor tissue was prepared by frozen section. It was treated in 0.2 mol/L HCl at room temperature for 15 min, incubated with 0.25% pepsin at 37°C for 30 min, and fixed with 4% paraformaldehyde for 20 min. Hybridization solution was added, and the mixture was incubated at 55°C for 2 h. The probe was diluted with the hybrid solution at a ratio of 1 : 200. Denaturation was carried out at 85°C for 2 min on the PCR instrument, and equilibrium was carried out at 37°C for 2 min. Afterward, the probe was added to the PCR apparatus and incubated overnight at 37°C under dark conditions. DAPI was added for redyeing for 20 min, antifluorescence quench agent was used to seal the tablets, and fluorescence microscope was used to take photos.

### 2.8. Double Luciferase Reporter Gene

StarBase predicted that the 3′UTR of LINC01087 and PPM1E contains the complementary sequence of miR-135a-5p. Gene mutation technology was used to mutate the binding site. The sequences containing the mutation and binding site were inserted into the luciferase reporter gene vector. Then, the wild-type vector WT-PPM1E and mutant vector MUT-PPM1E were constructed. psiCHECK-2-LINC01087-3′UTR or psiCHECK-2-LINC01087-3′UTR-MUT was constructed. A total of 20 ng plasmid was co-transfected with miR-135a-5pmimic or NC into cells and detected using the luciferase dual luciferase reporter system (Promega, USA).

### 2.9. Statistical Analysis

Statistical analysis was performed using SPSS 21.0 software. The data were expressed as mean ± standard deviation. The independent sample *t*-test was used for comparison between the two groups. One-way analysis of variance was used for comparison between multiple groups. *p* < 0.05 was considered statistically different.

## 3. Results

### 3.1. Expression of LINC01087 in Thyroid Cancer

**Figure 1:**
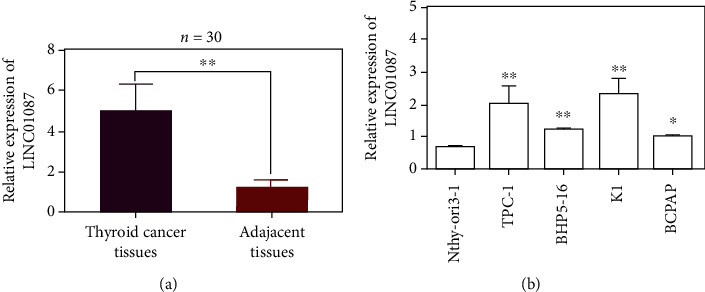
Identification and screening of LINC01087. (a) The expression level of LINC01087 in 60 pairs of thyroid cancer tissues and adjacent tissues. (b) Expression of LINC01087 in four PTC cell lines (TPC-1, BHP5-16, K1, and BCPAP) and normal human thyroid epithelial cell line Nthy-ori3-1. ^∗^*p* < 0.05, ^∗∗^*p* < 0.01.

LINC01087 was highly expressed in thyroid cancer tissues (5.077 ± 1.267) compared with adjacent control tissues (1.250 ± 0.379, *n* = 30, Figure 1(a), *p* < 0.01). In comparison with Nthy-ori3-1 cells, the LINC01087 expression level in TPC-1, K1, BHp5-16, and BCPAP increased (Figure 1(b), *p* < 0.05). LINC01087 is highly expressed in thyroid carcinoma.

### 3.2. Knockdown LINC01087 on EMT of Thyroid Cancer Cells

**Figure 2:**
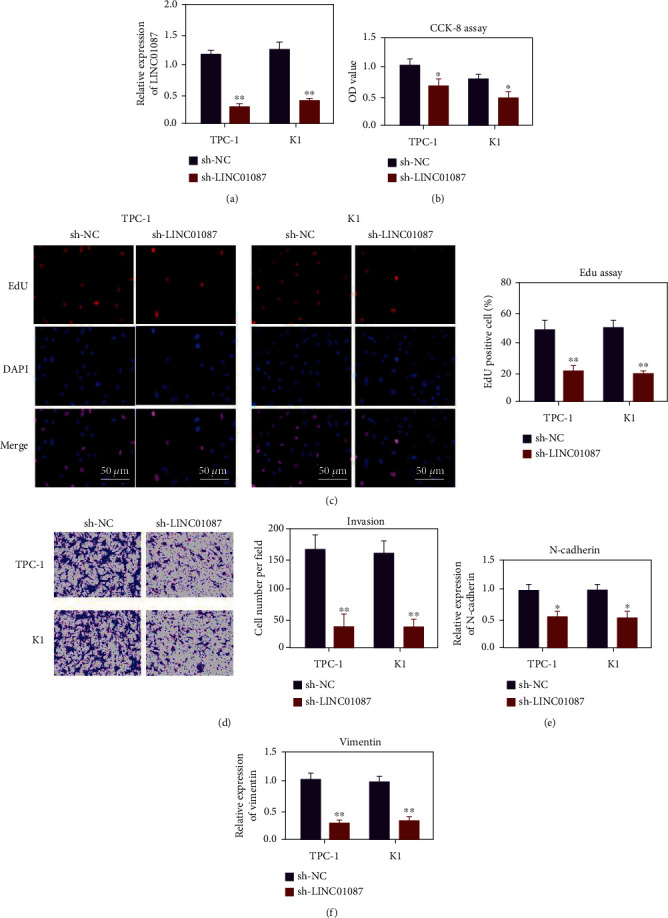
LINC01087 can promote the proliferation, migration, and invasion of thyroid cancer cells. (a) Knockout efficiency of plasmid shNC and sh-LINC01087 transfected into thyroid cancer cells. (b) CCK-8 detects the effect of LINC01087 knockdown on the proliferation of TPC-1 and K1 cells. (c) The EdU method was used to evaluate the effect of LINC01087 knockdown on the proliferation of TPC-1 and K1 cells. (d) Evaluate the effect of LINC01087 gene knockdown on the invasion ability of TPC-1 and K1 cells through the transwell invasion experiment. (e) After knocking down LINC01087, QRT-PCR detects the expression of N-cad. (f) After knocking down LINC01087, QRT-PCR detects the expression of vimentin. ^∗^*p* < 0.05, ^∗∗^*p* < 0.01.

In comparison with the sh-NC group, the expression level of LINC01087 in the sh-LINC01087 group decreased (Figure 2(a), *p* < 0.01). Cell proliferation test results showed that the proliferation ability of TPC-1 and K1 cells decreased when LINC01087 was knocked down (Figures 2(b) and 2(c), *p* < 0.05). Transwell results also showed that the migration and invasion abilities of TPC-1 and K1 cells were reduced after LINC01087 knockdown (Figure 2(d), *p* < 0.01). qRT-PCR results showed that after LINC01087 knockdown, N-cadherin and vimentin were decreased (Figures 2(e) and 2(f), *p* < 0.05).

### 3.3. LINC01087 Targets the miR-135a-5p Expression

**Figure 3:**
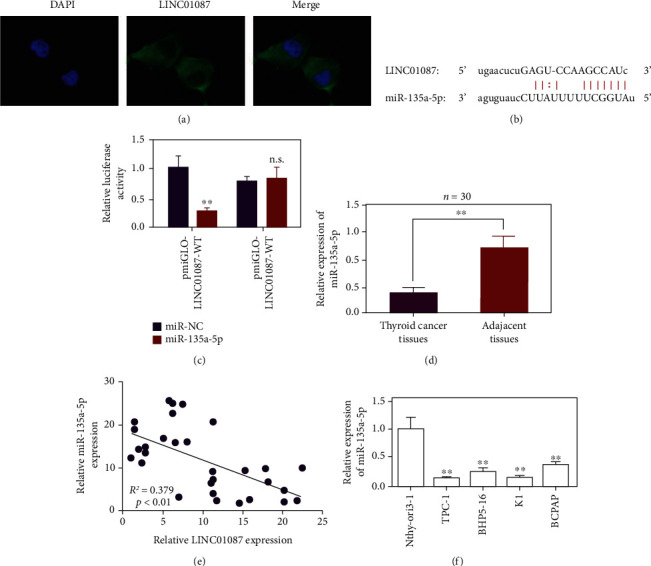
Determination of hsa-miR-135a-5p as the target gene of LINC01087. (a) Determine the location of LINC01087 in thyroid cancer cells by FISH method. (b) Schematic diagram of the binding site of wild-type LINC01087 and hsa-miR-135a-5p. (c) LINC01087 directly binds to hsa-miR-135a-5p, verified by dual fluorescein reporter gene test. (d) qPCR to detect the expression of hsa-miR-135a-5p in thyroid cancer tissues and adjacent tissues. (e) Pearson correlation analysis of the correlation between hsa-miR-135a-5p and LINC01087 in thyroid cancer. (f) Expression of hsa-miR-135a-5p in four PTC cell lines (TPC-1, BHP5-16, K1, and BCPAP) and normal human thyroid epithelial cell line Nthy-ori3-1. ^∗∗^*p* < 0.01.

The localization of LINC01087 in the cytoplasm of thyroid cancer cells was determined using the FISH method (Figure 3(a)). The existence of binding sites between LINC01087 and miR-135a-5p was predicted using the StarBase online software (Figure 3(b)). After transfection with wild-type LINC01087 expression vector, the luciferase activity of thyroid cancer cells in the miR-135a-5p group decreased compared with the miR-Con group. No signal changed after transfection with mutant LINC01087 expression vector (Figure 3(c), *p* < 0.01). miR-135a-5p was underexpressed in thyroid cancer tissues (0.369 ± 0.130 in cancer and 1.066 ± 0.121 in adjacent tissues, Figure 3(d), *p* < 0.01). The person correlation analysis reveals that the expressions of miR-135a-5p and LINC01087 are correlated in thyroid cancer. Hsa-miR-135a-5p and LINC01087 were negatively correlated with a correlation coefficient of –0.379 (Figure 3(e), *p* < 0.01). In comparison with Nthy-ori3-1 cells, miR-135a-5p in TPC-1, K1, BHp5-16, and BCPAP were reduced (Figure 3(f), *p* < 0.01).

### 3.4. miR-135a-5p Inhibited Ovarian Cancer Cells TPC-1 and K1

**Figure 4:**
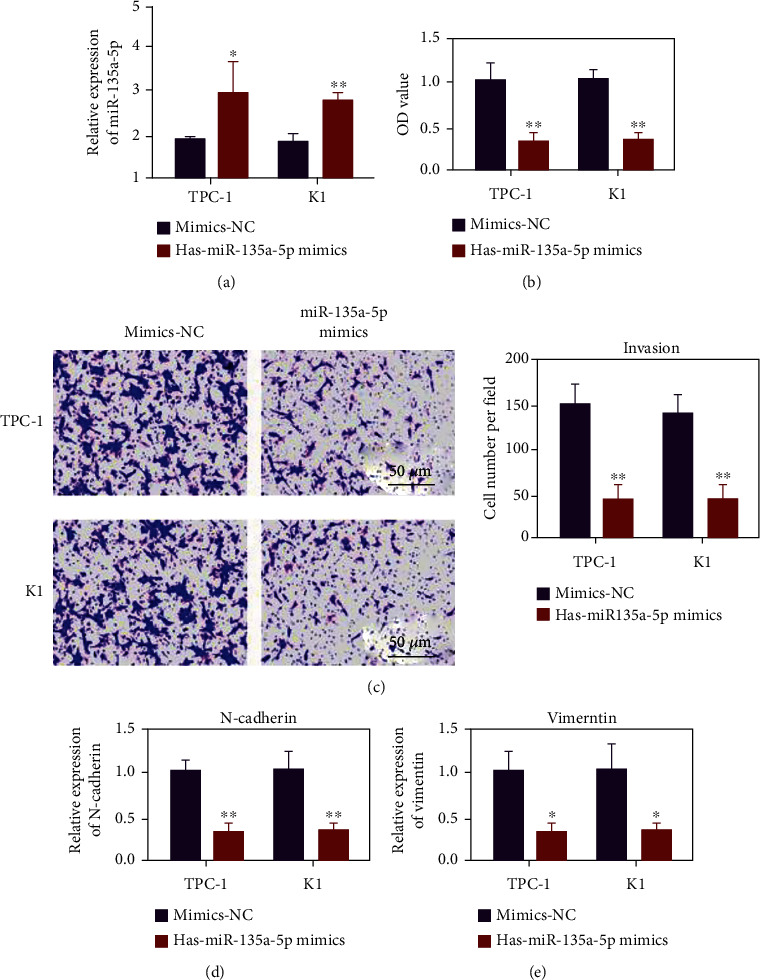
Hsa-miR-135a-5p can inhibit the proliferation, migration, and invasion of thyroid cancer cells. (a) Detection of miR-135a-5p transfection efficiency. (b) Evaluation of the effect of adding hsa-miR-135a-5p mimics on the proliferation of TPC-1 and K1 cells by CCK-8. (c) Evaluate the effect of the addition of hsa-miR-135a-5p mimics on the invasion ability of TPC-1 and K1 cells through the Transwell invasion experiment. (d) After adding hsa-miR-135a-5p mimics, qRT-PCR was used to detect the expression of N-cad. (e) After adding hsa-miR-135a-5p mimics, use qRT-PCR to detect the expression of vimentin. ^∗^*p* < 0.05, ^∗∗^*p* < 0.01.

The expression of miR-135a-5p in the mimic group increased (Figure 4(a), *p* < 0.05). In comparison with the mimic-NC group, the cell activity of the miR-135a-5p mimic group decreased. The overexpression of miR-135a-5p can inhibit the proliferation of TPC-1 and K1 (Figure 4(b), *p* < 0.05). Transwell experimental results show that the overexpression of miR-135a-5p could inhibit the invasion ability of TPC-1 and K1 cells (Figure 4(c), *p* < 0.01). RNA was extracted after transfection of miR-135a-5p mimics, and expression levels of N-cadherin and vimentin were detected by QRT-PCR. The overexpression of miR-135a-5p decreased the expression of N-cadherin and vimentin (Figures 4(d) and 4(e), *p* < 0.05).

### 3.5. miR-135a-5p Regulates the Expression of PPM1E

**Figure 5:**
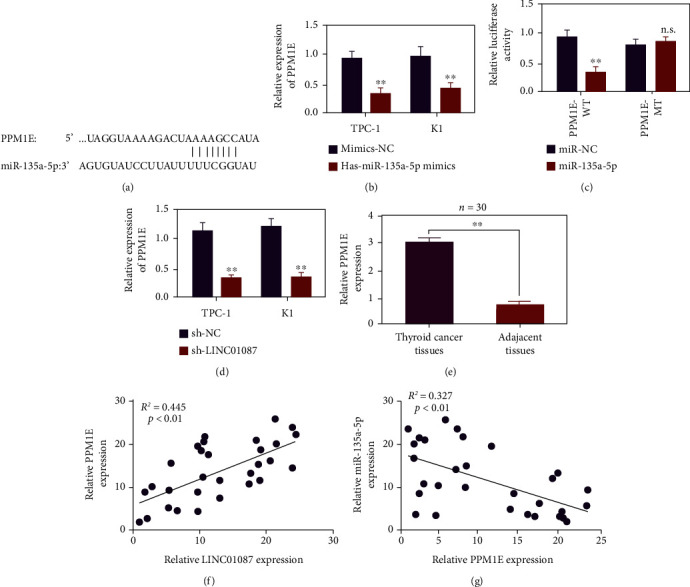
LINC01087 regulates PPM1E through hsa-miR-135a-5p. (a) Schematic diagram of the binding site of wild-type PPM1E and hsa-miR-135a-5p. (b) qPCR detection of the PPM1E expression after adding hsa-miR-135a-5p mimic. (c) Verify the binding of hsa-miR-135a-5p and PPM1E through dual fluorescein reporter gene test. (d) Detect the expression of PPM1E after LINC01087 knockdown by qPCR. (e) Use qPCR to detect the expression level of PPM1E in thyroid cancer tissues and adjacent tissues. (f) Use Pearson correlation to analyze the correlation between PPM1E and hsa-miR-135a-5p in thyroid cancer. ^∗∗^*p* < 0.01.

TargetScan prediction a targeted binding site between miR-135a-5p and PPM1E (Figure 5(a)). In comparison with the miR-135a-5p group, the protein level of PPM1E in the miR-135a-5p group significantly decreased (Figure 5(b), *p* < 0.01). The results of the double luciferase report show that the luciferase activity of the miR-135a-5p group decreased in the cell experiment of the wild-type carrier WT-PPM1E compared with miR-Con. In the cell experiment of the mutated carrier MUT-PPM1E, no significant difference was observed in the luciferase activity between the miR-135a-5p-5p and miR-Con group (Figure 5(c), *p* < 0.01). In compared with the sh-Con group, the protein level of PPM1E in the sh-LINC01087 group decreased (Figure 5(d), *p* < 0.01). In comparison with the adjacent control tissues, PPM1E was highly expressed in thyroid cancer tissues (*n* = 30, Figure 5(e), *p* < 0.01). Pearson correlation analysis showed that PPM1E and miR-135a-5p are negatively correlated in thyroid cancer with correlation coefficient of –0.327 (Figure 5(f)).

### 3.6. Overexpression of PPM1E Partially Reversed the Inhibitory Effect of miR-135a-5p on the Proliferation and Invasion of HUMAN Ovarian Cancer Cells TPC-1 and K1

**Figure 6:**
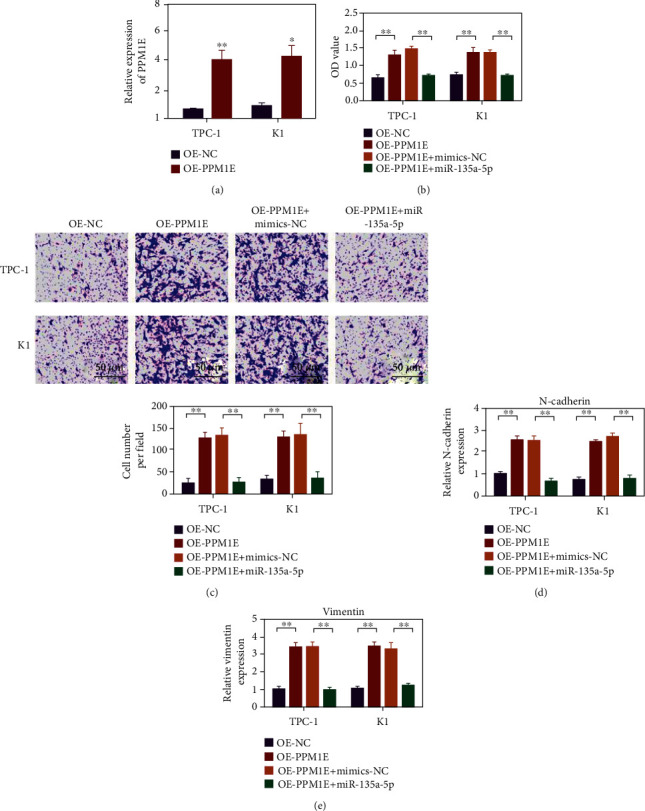
has-miR-135a-5p inhibits the proliferation, migration, and invasion of thyroid cancer by targeting PPM1E. (a) Use qPCR to detect the expression level of PPM1E in TPC-1 and K1 cells. (b) Using the CCK-8 method to evaluate the effects on the proliferation of TPC-1 and K1 cells under different conditions. (c) Transwell invasion experiment evaluates the influence of different conditions on the invasion ability of TPC-1 and K1 cells. (d) qRT-PCR detects the expression of N-cad in TPC-1 and K1 cells under different conditions. (e) qRT-PCR detects the expression of vimentin in TPC-1 and K1 cells under different conditions. ^∗^*p* < 0.05, ^∗∗^*p* < 0.01.

Figure 6(a) shows that the overexpressed plasmid upregulated the expression of PPM1E in ovarian cancer cells. In comparison with the OE-NC group, the OE-PPM1E group had higher cell activity and more invasive cells. In comparison with the OE-PPM1E+ MITIS-NC group, the cell viability and invasive cell number significantly decreased in the OE-PPM1E+ miR-135a-5p group (Figures 6(b) and 6(c), *p* < 0.01). N-cadherin and vimentin increased in the OE-PPM1E group. In comparison with the OE-PPM1E+ mimic-NC group, N-cadherin and vimentin expression levels decreased in the OE-PPM1E+ miR-135a-5p group (Figures 6(d) and 6(e), *p* < 0.01).

### 3.7. Overexpression of PPM1E Partially Reversed the Inhibitory Effect of LINC01087 on the Proliferation and Invasion of HUMAN Ovarian Cancer Cells TPC-1 and K1

**Figure 7:**
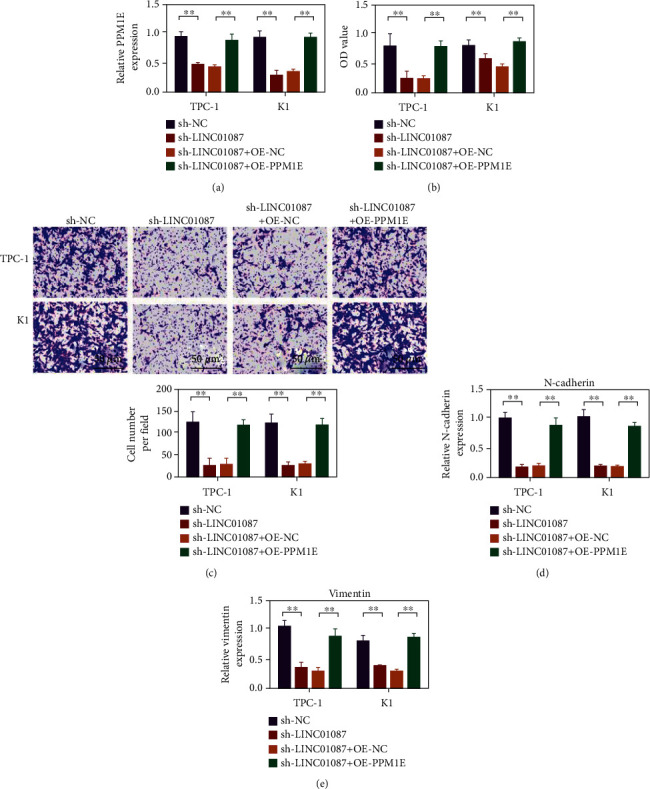
The PPM1E overexpression can reverse the inhibitory effect of LINC01087 gene knockdown on the proliferation, migration, and invasion of thyroid cancer cells. (a) Use qPCR to detect the expression level of PPM1E in TPC-1 and K1 cells. (b) CCK-8 experiment to evaluate the effects of different conditions on the proliferation of TPC-1 and K1 cells. (c) The transwell invasion experiment was used to evaluate the influence of different conditions on the invasion ability of TPC-1 and K1 cells. (d) Using the qRT-PCR method to detect the expression of N-cad in TPC-1 and K1 cells under different conditions. (e) Using the qRT-PCR method to detect the expression of vimentin in TPC-1 and K1 cells under different conditions. ^∗∗^*p* < 0.01.

As shown in Figure 7(a), the expression of PPM1E decreased after LINC01087 was silenced. The expression of PPM1E was upregulated in the sh-LINC01087 + OE-PPM1E cotransfection group. In comparison with the sh-NC group, the cell activity of sh-LINC01087 group and the number of invaded cells decreased. In comparison with the sh-LINC01087 + OE-NC group, the cell viability of the sh-LINC01087 + OE-PPM1E group and the number of invasive cells significantly increased (Figures 7(b) and 7(c), *p* < 0.01). N-cadherin and vimentin decreased in the sh-LINC01087 group. In comparison with the sh-LINC01087 + OE-NC group, N-cadherin and vimentin were upregulated in the sh-LINC01087 + OE-PPM1E group (Figures 7(d) and 7(e), *p* < 0.01).

## 4. Discussion

Molecular diagnosis of cancer and gene targeted therapy has been the focus of recent research [19–21]. The expression levels of some lncRNAs in tumor cells were changed [22, 23]. The lncRNA expression is dysregulated in thyroid cancer [24–26]. lncRNAs play an important role in the development of thyroid cancer. Braf activated noncoding RNA (BANCR), a 693-bp nucleotide transcript, was first identified in melanoma. Zheng et al. [27, 28] found that BANCR is highly expressed in human thyroid tumor tissues. BANCR knockdown inhibits the cell proliferation by downregulating cyclin D1. Zhou et al. [28] analyzed the PVT1 expression in 84 thyroid cancer. Results showed that the PVT1 expression was higher in thyroid cancer, and PVT1 silencing decelerated cell growth. H19 gene, which is located on human chromosome 11P15, is an imprinted oncoembryo gene. MALAT1 disorders are involved in regulating cell cycle, proliferation, and migration various cancers. Huang et al. [29] detected high levels of the MALAT1 expression in thyroid cancer cell lines and FTC tissues. MALAT1 can upregulate the IQGAP1 expression. HOTAIR is upregulated in thyroid carcinoma compared with normal thyroid tissue [30, 31].

This study investigated the expression of LINC01087 in thyroid cancer. The biological role and downstream regulatory mechanism of LINC01087 in thyroid cancer was further studied. The expression level of LINC01087 in thyroid carcinoma and paracarcinoma tissues was compared by SYBR green real-time fluorescence quantitative PCR. LINC01087 was highly expressed in thyroid carcinoma tissues, and its expression was higher than that in paracarcinoma tissues. Results indicate that the high expression of LINC01087 can be used as a specific marker of thyroid cancer and has important value for the early diagnosis of thyroid cancer.

The results of this study showed that miR-135a-5p was underexpressed in thyroid cancer cells, and the overexpression of miR-135a-5p inhibited the expression of PPM1E protein. The overexpression of miR-135a-5p also inhibited the proliferation of TPC-1 and K1 in thyroid cancer cells. Moreover, miR-135a-5p was targeted by LINC01087. The low expression of miR-135a-5p partially reversed the inhibitory effect of LINC01087 on TPC-1 and K1 cell proliferation and that of LINC01087 on PPM1E.

PP2C is an important class of protein phosphatases in eukaryotes [32, 33]. PP2C negatively regulates signal transduction pathways in vivo by catalyzing the dephosphorylation of substrate proteins [34]. The discovery of reversible protein phosphorylation is a milestone in the research of cell signal transduction. Protein kinases and phosphatases are key enzymes that catalyze reversible protein phosphorylation and dephosphorylation [35]. This modification at the posttranslational level can alter the physiological and biochemical properties of many key proteins in the signaling pathway, thus affecting cell phenotype. In the present study, LINC01087 knockdown inhibited the EMT of TPC-1 and K1. The expression of PPM1E was inhibited by LINC01087 knockdown. In addition, the low expression of miR-135a-5p reversed the effect of the low expression of LINC01087 on PPM1E. Therefore, LINC01087 may regulate the expression of PPM1E through miR-135a-5p, thereby affecting the proliferation and metastasis of thyroid cancer cells.

## 5. Conclusion

In conclusion, LINC01087 affects the development and progression of thyroid cancer. Early diagnosis of thyroid cancer has been the focus of recent research. This study provides some implications for the treatment of thyroid cancer. However, this study may have some defects, and further studies should focus on the mechanism of LINC01087's involvement in the EMT of thyroid cancer.

## Figures and Tables

**Table 1:** Gene primer sequence.

GENEs	Upstream sequence (5′-3′)	Downstream sequence (5′-3′)
LINC01087	ATGTCCTTTACCCCAAGAATCAT	ACAAGCCTTTTCCTAGGCTGTA
miR-135a-5p	GGCCTCGCTGTTCTCTATGG	GCCACGGCTCCAATCCCTAT
PPM1E	GGACTGTGGGACCAGGAAGAAC	CACAAACCGCTCATCAGTGAC
*β*-Actin	TTTTGGCTATACCCTACTGGCA	CTGCACAGTCGTCAGCATATC

## Data Availability

The data used to support the findings of this study are available from the corresponding author upon request.

## References

[1] Brito J. P., Hay I. D., Morris J. C. (2014). Low risk papillary thyroid cancer. *BMJ*.

[2] Nix P., Nicolaides A., Coatesworth A. (2005). Thyroid cancer review 1: presentation and investigation of thyroid cancer. *International Journal of Clinical Practice*.

[3] Nikiforov Y. E., Carty S. E., Chiosea S. I. (2014). Highly accurate diagnosis of cancer in thyroid nodules with follicular neoplasm/suspicious for a follicular neoplasm cytology by ThyroSeq v2 next-generation sequencing assay. *Cancer*.

[4] Kim J., Gosnell J. E., Roman S. A. (2020). Geographic influences in the global rise of thyroid cancer. *Nature Reviews Endocrinology*.

[5] Li M., Dal Maso L., Vaccarella S. (2020). Global trends in thyroid cancer incidence and the impact of overdiagnosis. *The Lancet Diabetes & Endocrinology*.

[6] Raty J., Pikkarainen J., Wirth T., Yla-Herttuala S. (2008). Gene therapy: the first approved gene-based medicines, molecular mechanisms and clinical indications. *Current Molecular Pharmacology*.

[7] Hardin H., Helein H., Meyer K. (2018). Thyroid cancer stem-like cell exosomes: regulation of EMT via transfer of lncRNAs. *Laboratory Investigation*.

[8] Liang W., Sun F. (2019). Identification of pivotal lncRNAs in papillary thyroid cancer using lncRNA–mRNA–miRNA ceRNA network analysis. *PeerJ*.

[9] Lei H., Gao Y., Xu X. (2017). LncRNA TUG1 influences papillary thyroid cancer cell proliferation, migration and EMT formation through targeting miR-145. *Acta Biochimica et Biophysica Sinica*.

[10] Zhang Y., Yang W. Q., Zhu H. (2014). Regulation of autophagy by miR-30d impacts sensitivity of anaplastic thyroid carcinoma to cisplatin. *Biochemical Pharmacology*.

[11] She J.-K., Fu D. N., Zhen D., Gong G. H., Zhang B. (2020). LINC01087 is highly expressed in breast cancer and regulates the malignant behavior of cancer cells through miR-335-5p/Rock1. *Oncotargets and Therapy*.

[12] Chen W., Wang F., Zhang J., Li C., Hong L. (2022). LINC01087 indicates a poor prognosis of glioma patients with preoperative MRI. *Functional & Integrative Genomics*.

[13] Ramírez-Moya J., Santisteban P. (2019). miRNA-directed regulation of the main signaling pathways in thyroid cancer. *Frontiers in Endocrinology*.

[14] Dai D., Tan Y., Guo L., Tang A., Zhao Y. (2020). Identification of exosomal miRNA biomarkers for diagnosis of papillary thyroid cancer by small RNA sequencing. *European Journal of Endocrinology*.

[15] Shan H., Guo D., Zhang S. (2020). SNHG6 modulates oxidized low-density lipoprotein-induced endothelial cells injury through miR-135a-5p/ROCK in atherosclerosis. *Cell & Bioscience*.

[16] Chen H., Li X. (2019). Withdrawn: LncRNA-ROR is involved in cerebral hypoxia/reoxygenation-induced injury via regulating miR-135a-5p/ROCK1/2. *American Journal of Translational Research*.

[17] Ma S., Gu X., Shen L. (2021). CircHAS2 promotes the proliferation, migration, and invasion of gastric cancer cells by regulating PPM1E mediated by hsa-miR-944. *Cell Death & Disease*.

[18] Voss M., Paterson J., Kelsall I. R. (2011). Ppm1E is an in cellulo AMP-activated protein kinase phosphatase. *Cellular Signalling*.

[19] Olson E., Wintheiser G., Wolfe K. M., Droessler J., Silberstein P. T. (2019). Epidemiology of thyroid cancer: a review of the National Cancer Database 2000-2013. *Cureus*.

[20] Onyango P., Feinberg A. P. (2011). A nucleolar protein, H19 opposite tumor suppressor (HOTS), is a tumor growth inhibitor encoded by a human imprinted H19 antisense transcript. *Proceedings of the National Academy of Sciences*.

[21] Autuoro J. M., Pirnie S. P., Carmichael G. G. (2014). Long noncoding RNAs in imprinting and X chromosome inactivation. *Biomolecules*.

[22] Santarpia L., Calin G. A., Adam L. (2013). A miRNA signature associated with human metastatic medullary thyroid carcinoma. *Endocrine-Related Cancer*.

[23] Yang M., Lu H., Liu J., Wu S., Kim P., Zhou X. (2022). lncRNAfunc: a knowledgebase of lncRNA function in human cancer. *Nucleic Acids Research*.

[24] Zhang X., Li D., Jia C., Cai H., Lv Z., Wu B. (2021). METTL14 promotes tumorigenesis by regulating lncRNA OIP5-AS1/miR-98/ADAMTS8 signaling in papillary thyroid cancer. *Cell Death & Disease*.

[25] Du Y.-L., Liang Y., Cao Y., Liu L., Li J., Shi G. Q. (2021). LncRNA XIST promotes migration and invasion of papillary thyroid cancer cell by modulating MiR-101-3p/CLDN1 axis. *Biochemical Genetics*.

[26] Li C., Chen X., Liu T., Chen G. (2021). lncRNA HOTAIRM1 regulates cell proliferation and the metastasis of thyroid cancer by targeting Wnt10b. *Oncology Reports*.

[27] Zheng H., Wang M., Jiang L. (2016). BRAF-activated long noncoding RNA modulates papillary thyroid carcinoma cell proliferation through regulating thyroid stimulating hormone receptor. *Cancer Research and Treatment: Official Journal of Korean Cancer Association*.

[28] Zhou Q., Chen J., Feng J., Wang J. (2016). Long noncoding RNA PVT1 modulates thyroid cancer cell proliferation by recruiting EZH2 and regulating thyroid-stimulating hormone receptor (TSHR). *Tumor Biology*.

[29] Huang J. K., Ma L., Song W. H. (2016). MALAT1 promotes the proliferation and invasion of thyroid cancer cells via regulating the expression of IQGAP1. *Biomedicine & Pharmacotherapy*.

[30] Zhu H., Lv Z., An C. (2016). Onco-lncRNA _HOTAIR_ and its functional genetic variants in papillary thyroid carcinoma. *Scientific Reports*.

[31] Li H. M., Yang H., Wen D. Y. (2017). Overexpression of LncRNA HOTAIR is associated with poor prognosis in thyroid carcinoma: a study based on TCGA and GEO data. *Hormone and Metabolic Research*.

[32] Chen M.-B., Liu Y. Y., Cheng L. B., Lu J. W., Zeng P., Lu P. H. (2017). AMPK*α* phosphatase Ppm1E upregulation in human gastric cancer is required for cell proliferation. *Oncotarget*.

[33] Ma Y., Szostkiewicz I., Korte A. (2009). Regulators of PP2C phosphatase activity function as abscisic acid sensors. *Science*.

[34] Soon F.-F., Ng L. M., Zhou X. E. (2012). Molecular mimicry regulates ABA signaling by SnRK2 kinases and PP2C phosphatases. *Science*.

[35] Bork P., Brown N. P., Hegyi H., Schultz J. (1996). The protein phosphatase 2C (PP2C) superfamily: detection of bacterial homologues. *Protein Science*.

